# Hysteretic slit-snapping and multistability in buckled beams with partial cuts

**DOI:** 10.1126/sciadv.aeb9750

**Published:** 2026-02-13

**Authors:** Bernat Durà Faulí, Lennard Kwakernaak, Martin van Hecke

**Affiliations:** ^1^ AMOLF, Science Park 104, 1098 XG Amsterdam, Netherlands.; ^2^ Huygens-Kamerlingh Onnes Laboratory, Universiteit Leiden, PO Box 9504, 2300 RA Leiden, Netherlands.

## Abstract

Elastic instabilities such as buckling and snapping have evolved into a powerful design principle, enabling memory, sequential shape morphing, and computing in metamaterials and devices. Modifying the postbuckling configurations or their snapping transitions would greatly expand design possibilities, yet general principles for controlling elastic instabilities are lacking. Here, we show that adding a partial cut, or slit, to a flexible beam enables precise control of postbuckling behavior: Under compression, slit-beams first buckle and then snap, leading to tristability within the hysteretic regime. A truss model explains these phenomena by uncovering the interplay of geometric and slit-induced nonlinearities. Leveraging these insights, we realize multislit beams with programmable behavior, unlocking a vast design space featuring giant hysteresis, quadstability, multistep snapping, tristability at zero compression, and compression-induced snapping between left- and right-buckled states. Our strategy is general, simple to design and implement, and enables mechanical metamaterials and devices with advanced memory and sequential behavior.

## Teaser

Partial cuts in buckling elastic beams create controllable snapping transitions and multiple stable shapes.

## INTRODUCTION

The main elastic instabilities are buckling, where an initially straight structure spontaneously curves under compression, and snapping, where a curved structure hysteretically changes shape under transverse loading (*1*). Underlying these instabilities are geometric nonlinearities, where large deformations of slender structures dominate their mechanical response (*2*, *3*). While instabilities are important to avoid in many industrial structures, they are explored in nature to power, e.g., the snapping of the Venus flytrap (*4*, *5*) or the jumping of locust and grasshoppers (*6*, *7*). This has inspired the use of buckling and snapping for applications such as rapid actuation, energy damping and harvesting, and switchable materials in soft robots and metamaterials (*8*–*15*). In particular, the bistability of beams in the postbuckling regime allows them to function as “material bits,” which can be explored for mechanical memory (*16*), sequential shape morphing (*17*, *18*), and computing (*19*, *20*).

Constraints on the instabilities, or the conditions under which they occur, thus limit the range of functionalities. One route to enlarge this range is using very wide beams with aspect ratios above 0.24, which can produce snapping and creasing, but to reach these regimes large strains and energies are required (*21*). However, for smaller aspect ratios, a rectangular beam under axial compression buckles but does not snap under increased compression; postbuckling, it takes on only two configurations; and multistability requires compression. While general principles to modify instabilities are lacking, in particular for altering the number of postbuckling branches or designing the snapping transitions between them, we note that contact interactions are another source of mechanical nonlinearities (*19*, *22*, *23*). This poses the question if and how the repertoire of elastic instabilities can be augmented by combing contact and geometric instabilities within a single slender structure.

Here, we show that endowing flexible structures with partial cuts, or slits, substantially expands their range of mechanical instabilities. Such slits straightforwardly introduce (contact) nonlinearities: While compressive stresses keep the slit closed and preserve the instabilities of the uncut structure, local tensile stresses can open the slit, effectively altering the geometry and enabling additional instabilities. While slits preserve the buckling instability associated with the closed structure, the curvature of buckled beams can induce tensile stresses that open the slits, making them ideally to control and modify the postbuckling behavior.

**Fig. 1.**
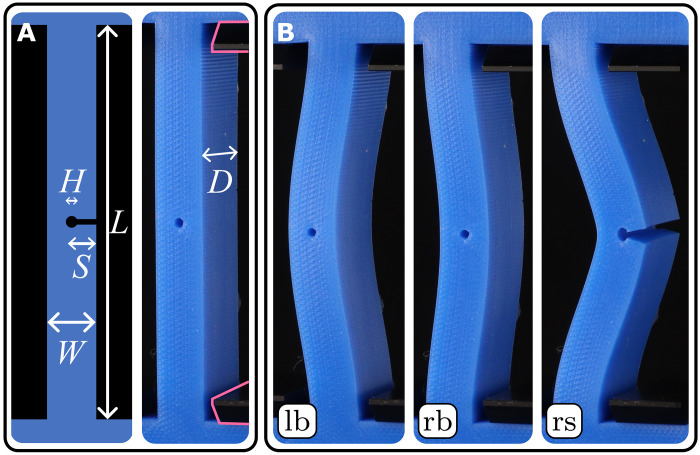
Slit beams: Buckling, snapping, and tristability. (**A**) Geometry of a slitted beam in its neutral configuration at zero compression. The beam is terminated with anchoring blocks (blue) and fixed by clamps (black, outline highlighted). (**B**) When the compressive strain ε is increased above the buckling strain ε_b_, the beam buckles and takes either a left-buckled (lb) or right-buckled (rb) configuration, both with closed slit. When ε is increased further, the right-buckled beam slit-snaps at ε_o_, and this right snapped (rs) configuration initially remains stable when the strain is decreased, until it snaps closed at ε_c_. The three panels evidence tristability in the hysteretic range ε_c_ < ε < ε_o_.

To illustrate this strategy, consider a straight beam with rectangular cross-section, modified with a transverse cut extending from the right edge to its midpoint (Fig. 1). When the compressive strain ε is increased, this slitted beam initially exhibits buckling, and the slit remains fully closed—inheriting the instability of the closed configuration which corresponds to an ordinary beam (Fig. 1C). Under increased compression, a right-buckled beam eventually exhibits a sudden slit-snapping instability that produces a strongly curved, open configuration (Fig. 1C). This instability is triggered by tensile stresses across the slit produced by the increasing curvature of postbuckling beams and leads to the switching from a closed to an open configuration under increased axial compression. As we detail below, the transition between open and closed configurations is hysteretic and leads to a tristable regime where the beams can be buckled to the left, buckled to the right, or right-snapped (Fig. 1C). The snapping and unsnapping of the beams defines the two critical strains ε_o_ and ε_c_. Hence, slits allows beams to both buckle and snap under compression and expand their multistability.

Here, we systematically explore the instabilities of slit beams, combining experiments, simulations, and theory. First, we demonstrate how the thickness of the beam and the size and position of the slit can be used to precisely control the opening and closing transitions. We capture the underlying mechanisms of slit-snapping and its parameter dependencies in an effective truss model, by examining its energy landscape. This naturally combines the bifurcation scenarios for closed and opened beams with the contact nonlinearities of the slit. We then use our insights obtained for single-slit beams to explore and rationally design beams with multiple slits and more exotic instabilities, where changes in the curvature induced by the opening or closing of one slit lead to interactions between the instabilities of the slits. For beams with dual slits, we determine the range of slit-snapping scenarios as function of slit location and experimentally realize beams with giant hysteresis, quadstability, and two-step snapping. Last, to further show the power of our strategy, we realize two multiple-slit beams with targeted extreme behavior: one that is tristable at zero compression and one that snaps between left and right buckling branches under compression. Our simple, geometric, and general strategy is poised to have a broad impact on the control and optimization of elastic instabilities, opening up exciting possibilities for applications in soft robotics, sensors, metamaterials, and in-material mechanical computing (*19*, *24*–*26*).

## RESULTS

### Experiments

We consider rectangular beams (length *L* = 80 mm, depth *D* = 25 mm, and variable width *W*), cast from Smooth-On Mold Star 30 VPS rubber in three-dimensional (3D) printed open-face molds (Fig. 1A). A transversal slit (length *S*) is cut after demolding using a custom-made guillotine and is terminated with a small hole (diameter *H* = 2 mm) that prevents tearing. We stress that neither the presence of the slit nor repeated snapping lead to the formation of cracks or aging of the sample—see the Supplementary Materials. We characterize slitted beams by the dimensionless parameters λ = *W*/*L* and *s* = *S*/*W*. The beams are mounted in a custom-made compression device that controls the uniaxial compressive strain ε ≔ *u*_*y*_/*L* by a stepper motor (strain rate, 2 mm/min) and records the configuration and dimensionless lateral midpoint deflection *x*_m_ = *x*/*L* by tracking white painted dots along the centerline of the beams (fig. S1).

### Bifurcation diagram

**Fig. 2.**
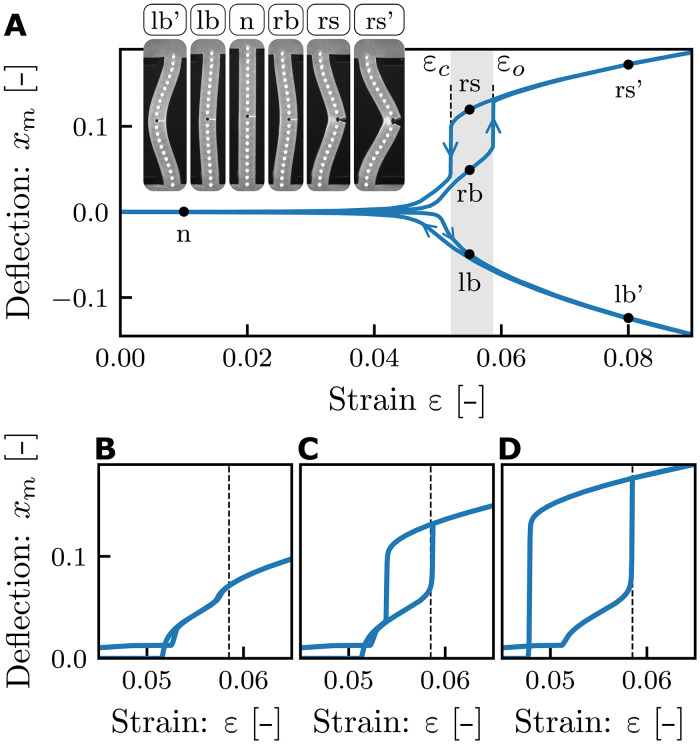
Bifurcation diagram and effect of slit size. (**A**) Bifurcation diagram observed in experiments, with configurations as indicated (λ = 0.123, *s* = 0.6, *H* = 2 mm). (**B** to **D**) Numerically obtained bifurcation diagrams for λ = 0.125 and (B) *s* = 0.3, (C) *s* = 0.6, and (D) *s* = 0.8. The opening strain ε_o_ = 0.0585 is independent of *s* (dashed), while the closing strain varies strongly with *s*.

We now consider the states and transitions for a slitted beam (λ = 0.123, *s* = 0.6, *H* = 2 mm) by slowly sweeping the compressive strain ε and characterizing the beam by the mid-beam deflection *x*_m_. This yields a bifurcation diagram that demonstrates both the tristability and snapping of slit beams (Fig. 2A and movies S1 and S2). At small strains, we observe ordinary buckling at ε ≈ 0.046. Right after this pitchfork bifurcation, the “lb” and “rb” configurations are mirror symmetric, and the slit remains closed (Figs. 1B and 2A). Further compression of the lb configuration yields a smooth and reversible increase of ∣*x*_m_∣. However, further compressing the rb configuration leads to a sharp snapping transition at the critical strain ε = ε_o_ ≈ 0.057, which results in a sharp increase of *x*_m_ and produces an open “rs” configuration. This transition is hysteretic: Lowering the strain yields an unsnapping transition from the open to the closed rb configuration at ε = ε_c_ ≈ 0.05. We refer to these phenomena as slit-snapping. In the regime ε_c_ < ε < ε_o_, the beam is tristable (Figs. 1C and 2A). Hence, the slit produces an additional solution branch of open configurations, which hysteretically connects to one of the ordinary buckling branches.

### Design parameters

We investigate the dependence of the bifurcation diagram and critical strains on the beam aspect ratio λ and slit size *s* by numerical simulations and by experiments. As expected, the aspect ratio λ sets the overall scale of the critical strains, which approximately scale as λ^2^, while the buckling strain ε_b_ remains independent of slit size (fig. S3). Considering the effect of slit size, we observe that a threshold of *s* ≈ 0.4 is necessary to initiate the opening transition. However, once snapping occurs, *s* no longer influences the opening strain ε_o_. In contrast, the slit size strongly affects the closing strain ε_c_ and thus controls the size of the hysteresis loop. This leads to three different slit-snapping scenarios: For very small *s*, the beam opens smoothly, developing in the *x*_m_(ε) curve a kink near ε_o_ (Fig. 2B); for intermediate *s*, we obtain the combined buckling-snapping bifurcation diagram with ε_c_ > ε_b_ (Fig. 2C). For large *s*, the snapped configuration remains stable below the buckling thresholds, so that ε_c_ < ε_b_ (Fig. 2D). The parameters λ and *s* enable the design of beams with tunable snapping transitions ε_o_ and ε_c_. The aspect ratio λ sets the scale of both ε_b_ and ε_o_, while *s* controls ε_c_ and the hysteresis width, which approaches a minimum of ε_c_ ≈ 0.5ε_o_ as *s* → 1. To design a slitted beam with prescribed values of ε_o_ and ε_c_, one should first select the beam aspect ratio λ, which determines ε_o_. Once λ is fixed, the slit length *s* can be increased to independently tune the hysteresis width, ε_o_ − ε_c_ and thereby achieve the desired ε_c_.

### Mechanism of slit-snapping

**Fig. 3.**
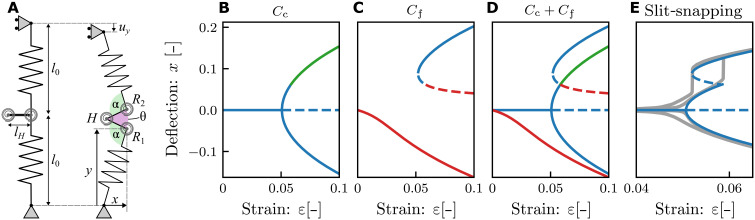
Slit-truss model. (**A**) Geometry at rest (left) and in the open state (right). The geometry and mechanics are specified by the truss lengths *l*_0_ and stiffnesses *k_s_*, lengths of the bars *l_H_*, and parameters of the center (left) hinge *H* (torsional stiffness κ_θ_, opening angle θ, rest angle zero) and right hinges *R*_1_ and *R*_2_ (torsional stiffness κ_α_, opening angle α, rest angle π/2). (**B** and **C**) Stable (full) and unstable (dashed) solutions of the slit-truss model. Here, we fix the parameters *l*_0_ = 1, *k*_s_ = 1, *l_H_* = 0.123, κ_α_ = 0.048, κ_θ_ = 2.54κ_α_. (B) Buckling for the closed (θ = 0) configurations *C*_c_. The right buckled branch includes a range where ∂*U*/∂θ > 0 (green), signaling tensile stresses that would open the slit; these solutions are not admissible. (C) Imperfect buckling for the free (θ unconstrained) *C*_f_ configurations. The snapped branches include regions where θ < 0 (red) which are not admissible. Notice that for finite compression, θ = 0 is reached at positive deflection *x*. (**D**) The combination of the free and constrained configurations captures the bifurcation diagram of slitted beams. (**E**) Zoom in, where the unstable closed (green) and self-overlapping open (red) branches are removed, and the solutions of the slitted-truss model (blue) are compared to experimental data (gray) for the beam in Fig. 2A.

To gain insight into the underlying mechanisms that governs slit-snapping, we develop a truss model based on the observations that slit beams exhibit buckling in their closed configuration and snap open when the stresses across the slit become tensile. We start from the Bellini truss—a minimal model for buckling—which consists of two equal linear springs of length *l*_o_, coupled by a torsional spring (*27*). To model a right-slit beam, the central Bellini hinge is replaced by a triplet of hinges connected by two additional bars, yielding a structure that breaks left-right symmetry (Fig. 3A). Its geometry is set by the length of the rigid bars *l*_H_ and the opening angle θ of the center hinge that encompasses both the closed (θ = 0) and open (θ > 0) configurations of a slit beam. The slit-truss system is connected at the bottom and top by two freely rotating joints and is compressed vertically by a strain ε = *u*_*y*_/(2*l*_0_). The compressed slit-truss structure remains top-down symmetric and is characterized by its mid-point deflection *x* and angle θ (Fig. 3A; for details, see the Supplementary Materials).

We model the configurations of the spring system by first solving for the closed (θ = 0) case, *C*_c_, and the free (θ unconstrained) case, *C*_f_, and then applying additional criteria derived from the physics of the slit. For the closed configuration *C*_c_, θ is fixed at zero, and the slit-truss model features an ordinary, perfect pitchfork bifurcation which represents the buckling of a closed beam (Fig. 3B). However, this solution is only admissible while it is energetically favorable for the slit to remain closed. To ensure that, we require for *C*_c_ that the unilateral constraint ∂*E*/∂θ ≤ 0 is satisfied, where *E* is the potential energy of the spring system. This eliminates part of the rb branch of *C*_c_ (Fig. 3B). In the free configuration, *C*_f_, the unconstrained truss model, due to the broken left-right symmetry, features an imperfect pitchfork bifurcation, producing a lb and a pair of stable and unstable rs solutions (Fig. 3C). However, enforcing the nonself-intersecting nature of the slit, these solutions are only admissible when θ ≥ 0, which eliminates parts of the lb and the unstable rs branch of the constrained system (Fig. 3D). Combining the admissible solutions produces the bifurcation diagram of slit-truss model, which closely matches the experimental observed behavior of slit beams (Fig. 3, D and E).

The solutions for the closed and free configurations and the admissibility conditions clarify the nature of the opening and closing transition of slit beams. The opening transition occurs at the intersection of the free and closed solutions, where θ, ∂*E*/∂*x*, and ∂*E*/∂θ all become zero. For larger strains, the closed solution turns unstable, and the configuration discontinuously snaps to the rs branch. Similarly, lowering the strain, the rs branch disappears in a simple saddle-node bifurcation, and the system discontinuously snaps closed. This description also clarifies the roles of the geometric and contact nonlinearities. The geometric nonlinearity of a closed beam controls the strain at which ∂*E*/∂θ changes sign and thus the opening strain. Moreover, the geometric nonlinearity of an open beam governs the saddle node bifurcation that sets the closing strain. The contact nonlinearity makes the system sensitive to the change in sign of ∂*E*/∂θ, thus enabling the opening transition.

The slit-truss model furthermore rationalizes the dependence of the opening and closing strains on the beam aspect ratio λ and slit size *s*. We note that the torsional constants of *R*_1_ and *R*_2_ can be determined by requiring that they correctly capture the onset of buckling in the *C*_c_ configurations, which implies that they scale as λ^2^ (*22*). In addition, κ_θ_ decreases for increasing *s*. In our model, the closing strain is controlled by the fold bifurcation of the free solution, and we find that, consistent with our experimental and numerical data, $\varepsilon_{c}^{\text{model}}$ decreases with decreasing *k*_θ_, allowing to establish a relation between *k*_θ_ and *s* that captures the three scenarios shown in Fig. 2 (fig. S5). Similarly, as the opening strain is determined by the onset of negative torques in the constraint solution, it is independent of κ_θ_ and thus *s*, consistent with our data. Hence, our model faithfully captures the phenomenology and leading parameter dependencies of slit-snapping.

### Beams with dual slits

The range and diversity of the snapping behavior of beams with a single slit is limited. First, snapping is restricted to a single pair of opening/closing transitions occurring for only one of the buckled branches. Furthermore, the hysteresis range is restricted, and the closing strain cannot be lowered below approximately 0.5ε_b_. Last, the snapping transition is unidirectional, always increasing the beam’s deflection.

**Fig. 4.**
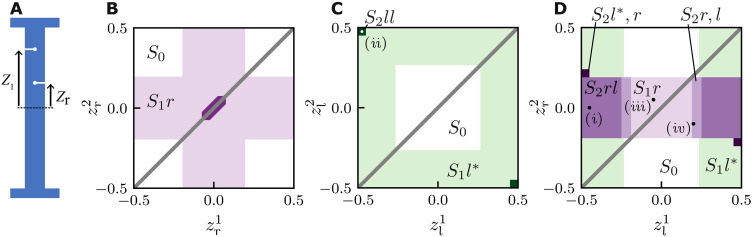
Enhanced snapping of dual slit beams. (**A**) Sketch of a beam with two slits, where *z*_r_ and *z*_l_ denote the slit positions of a right and left slit, respectively. (**B** to **D**) Snapping responses for beams with two right slits (B), a left and a right slit (C), and two left slits (D). The labels and colors denote eight qualitatively distinct behaviors. *S*_0_: No snapping. *S*_1_*r*: Single slit-snapping of a right slit. *S*_2_*rr*: Joint snapping of two right slits. *S*_2_*rl*: Cooperative snapping of a right and left slit. *S*_2_*r*, *l*: Sequential snapping (first right slit and then left slit). *S*_1_l*: Smooth opening of the left slit. *S*_2_l*, *r*: Snapping of the right slit triggered by previous smooth opening of the left slit. *S*_2_*ll*: Cooperative snapping of both left slits.

Here, we show that all these limitations can be overcome by using beams with multiple slits. We first explore the snapping of beams with two slits using finite element (FEM) simulations of beams with fixed thickness and slit depths (λ = 0.125, *s* = 0.75, *h* = 1 mm). As we will show, snapping of one slit drastically alters the curvature of the beam. Depending on the location of the slits, this can lead to “interactions” between the slits that are either cooperative, where one snapping event promotes the next, or antagonistic, where the first snapping event suppresses the second. We show numerically that these interactions give rise to a wide variety of slit-snapping scenarios (Fig. 4, B to D). We then experimentally realize dual-slit beams that materialize the most notable exotic snapping behaviors and lastly explore beams with more than two slits.

We parametrize the vertical positions of the right and left slits by the dimensionless variables $z_{r}^{i}$ and $z_{l}^{i}$, respectively, and examine the three possible arrangements of dual-slit beams: right-right, left-right, and left-left. For each, we discuss and classify the distinct scenarios as function of slit positions and the corresponding critical strains. Without loss of generality, we focus on the right-buckled branch; the left-buckled case follows by symmetry. Beams with two right slits exhibit three distinct regimes, which we label *S*_0_, *S*_1_*r*, and *S*_2_*rr* (Fig. 4B). Here, numerical indices indicate the number of slits that open, and “l” and “r” denote which slits participate. *S*_0_: No snapping occurs when both slits lie outside the center region, i.e., when $|z_{r}^{i}|>0.19$, analogous to the condition for a beam with a single slit (see the Supplementary Materials). *S*_1_*r*: Ordinary slit-snapping is observed whenever at least one slit is within the center region, i.e., has $z_{r}^{1}<0.19$. In this regime, the most central slit opens and prevents the other from doing so; the opening and closing strains match those of a single-slit beam. *S*_2_*rr*: When both $z_{r}^{i}\approx 0$, the slits cooperate and snap open and close simultaneously. The opening strain is identical to that of a single slit beam; the closing strain is slightly lower than that of a single slit beam. Hence, the rightward snapping of a beam with a pair of slits at the right side is quite similar to that of a single-slit beam.

In contrast, for left-left slits, right buckling beams exhibit three distinct behaviors (*S*_0_, *S*_1_l*, and *S*_2_*ll*). (Fig. 4C). *S*_0_: When the cuts are in the center region ($|z_{l}^{i}|<0.265$), the slits remain closed in right-buckling beams. *S*_1_l*: When one of the slits is located in the boundary region where the curvature is negative ($|z_{l}^{i}|<0.265$), it opens smoothly open but does not cause snapping. *S*_2_*ll*: Unexpectedly, there is a tiny parameter regime where, when both slits are close to the top and bottom boundaries of the beam, we observe right snapping with an opening strain ε_o_ lower than for a single slit at *z*_r_ = 0 (Fig. 4D). Apart from this exotic behavior, beams with left/left slits do not snap to the right.

Last, for beams with left-right slits, we observe a wide variety of snapping behaviors that we label *S*_2_*r*, *l*, *S*_2_*rl*, *S*_2_l*, *r*, and *S*_1_l*; here, a comma denotes sequential behavior, and stars indicate smooth opening of slits (Fig. 4D). We first focus on ∣*z*_r_∣ < 0.19 where three regimes are observed as a function of *z*_*l*_. *S*_1_*r*: When ∣*z*_l_∣ is small, we observe ordinary slit-snapping, controlled by *z*_r_—the left slit remains closed and the critical strains are as for a single slit beam. *S*_2_*r*, *l*: For intermediate ∣*z*_l_∣, we observe two-step snapping: After the right slit snaps open, increasing the compression leads to subsequent snapping of the left slit. Lowering the compression, both slits close sequentially, first the left and then the right. The right slit is the first to open and the last to close, and its critical strains are as for a beam with a single slit. *S*_2_*rl*: For extreme values of ∣*z*_l_∣ > 0.23, both slits open and close simultaneously and cooperatively. When the right slit opens, the resulting change in beam curvature immediately triggers the opening of the left slit; the opening strain is as for a single slit beam. However, the closing strain is lowered: The opening of the left slit prevents the right slit to close.

For large ∣*z*_r_∣ > 0.19, three additional regimes are found. *S*_0_: For small ∣*z*_l_∣ < 0.265, we observe no snapping. *S*_1_l*: For larger ∣*z*_l_∣, the left slit opens up continuously, and the right slit remains closed in most of parameter space. *S*_2_l*r: There is a small region of parameter space where the smooth opening of the left slit eventually triggers the snapping of the right slit. In this regime, both the opening and closing strains are modified from their single-slit values.

We cluster the snapping behavior of beams with two slits in three groups. First, in substantial swaths of parameter space, only one slit participates in the snapping behavior as the other remains closed (*S*_1_*r*). Second, in the regimes *S*_2_*rr*, *S*_2_*rl*, and *S*_2_*r*, *l*, the first snapping event is triggered by the instability of a single slit, and ε_o_ is as for single-slit beams. However, this initial event then triggers a subsequent snapping of the other slit, allowing to either lower ε_c_ or to realize sequential slit-snapping. Third, in extreme cases (*S*_2_l*, *r*, and *S*_2_*ll*), snapping is a cooperative effect and occurs for slit positions where a single slit would not produce snapping. Together, these results suggest that multiple slits allow to considerably extend the range of slit-snapping behaviors.

### Experimental realization of exotic snapping

**Fig. 5.**
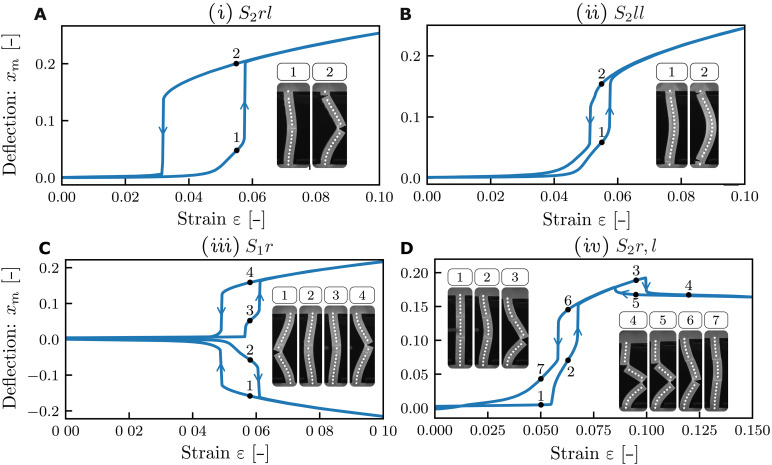
Experimental realization of four exotic slit-snapping scenarios in dual slit beams. Beam deflection versus strain with snapshots of distinct beam configurations. (**A**) Beam with large hysteresis range (regime *S*_2_*rl*, *z*_l_ = 0.45, and *z*_r_ = 0). (**B**) Beam with two left slits that snaps to the right (regime *S*_2_*ll* and *z*_l_ = −0.475, 0.475). Parameter values are also indicated in Fig. 4 as (i) to (iv). (**C**) Beam that snaps both to the left and to the right (regime *S*_1_*r*, *z*_l_ = −0.05, and *z*_r_ = 0.05). (**D**) Beam with sequential snapping and unsnapping (regime *S*_2_*r*, *l*, *z*_l_ = 0.2, and *z*_r_ = −0.1).

To demonstrate the potential of dual-slit beams experimentally, we realized the four most notable slit-snapping scenarios. To do so, we select four representative values for the slit positions based on the numerical simulations, fabricate the corresponding samples, and characterize their behavior (Fig. 5).

(i) First, we realize a beam with a large hysteresis loop in the *S*2*_rl_* regime, where a left slit near the beam’s end (*z*_l_ = 0.45) and a centered right slit (*z*_r_ = 0) act cooperatively to produce this response (Fig. 5A and movie S3). In essence, the increase in curvature induced by the snapping of the right slit at ϵ_o_ results in the simultaneous opening of the left slit, which in turn lowers the curvature of the top half of the beam (Fig. 5A). This lowered curvature suppresses the closing of the beam and strongly lowers the critical strain ε_c_. Hence, dual-slit beams can substantially enlarge the hysteresis range of slit beams.

(ii) Second, we fabricate a sample in the *S2ll* regime, where two left slits at the extremes of a beam, $\left(z_{l}^{1},z_{l}^{2}\right)=\left(0.475,-0.475\right)$, can cause rightward slit-snapping (Fig. 5B and movie S4). While the hysteresis loop of this sample resembles that of a single-slit beam, its postsnapping configuration is markedly different, lacking inflection points and resembling a buckled beam with pinned boundaries. Moreover, the snapping here requires both slits to be present, in contrast to case (i) where the snapping is triggered by the right slit independently of the presence of the left slit. Hence, scenario (iv) highlights that cooperation between slits can give rise to qualitatively distinct instabilities.

(iii) Third, we realize a quadstable beam, by using a left and a right slit to allow snapping of both the right and left buckled branches (Fig. 5C and movies S5 and S6). To select the slit positions, we note that point reflection symmetry of the design space about the origin implies that similar single–slit-snapping responses can be selected for both the left and right buckling branches by taking $z_{r}^{1}=-z_{l}^{2}$; here, we take a design inside the *S1* region [$\left(z_{r}^{1},z_{l}^{2}\right)=\left(0.05,-0.05\right)$]. When the beam buckles right (left), only the right (left) slit snaps, and the left (right) slit remains closed (Fig. 5C). Hence, this dual-slit beam can snap both to the left and to the right, mimicking the behavior of a single slit beam with either *z*_r_ = 0.05 or *z*_l_ = −0.05. We note that the slits do not cooperate, and this would allow to tune the snapping of their left and right buckled branch independently by, e.g., varying the corresponding slit depth.

(iv) Last, we realize sequential snapping with a beam in the *S2r,l* regime [$\left(z_{r}^{1},z_{l}^{2}\right)=\left(-0.1, 0.2\right)$; Fig. 5D and movie S7]. Under increasing strain, the beam initially exhibits regular snapping of the right slit while the left slit remains closed. As the strain increases further, the left slit snaps at a second snapping strain, leading to an S-shaped configuration in which both slits are open (Fig. 5D). Both snapping transitions are hysteretic, and as the strain is decreased, first the left and then the right slit closes. We note that in our experiments, the second slit closing leads to a small transversal mismatch between the top and bottom parts of the slit, making the smooth return to the unbuckled configuration hysteretic, as the mismatch is gradually disappears as the strain is removed (Fig. 5D). Hence, dual slits allow sequential snapping in a single beam.

Together, these four scenarios demonstrate the effectiveness of introducing an additional slit as a strategy to experimentally enhance and tune the snapping and response of slit beams. Moreover, the geometric understanding of slit-slit interactions provides a foundation for the rational design of targeted responses in beams with more than two slits, where exhaustive combinatorial exploration becomes impractical.

### Beams with many slits and extreme pathways

**Fig. 6.**
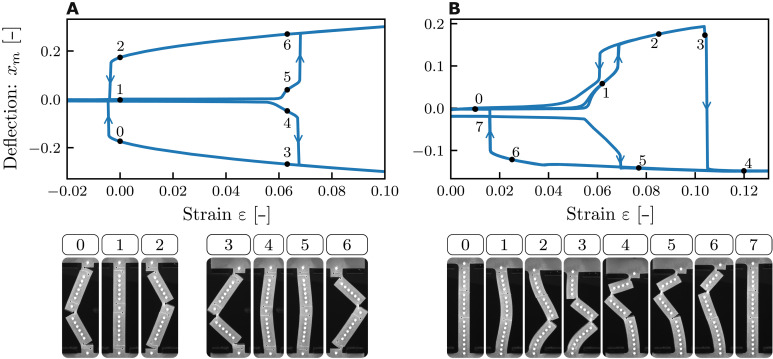
Complex snapping with multiple slits. (**A**) A beam with six deep slits (λ = 0.1365, *s* = 0.8, *z*_l_ = −0.45, −0.05, 0.4, *z*_r_ = −0.4, 0.05, 0.45) shows tristability at zero strain. (**B**) A beam with three slits (λ = 0.126, *s* = 0.75, *z*_l_ = 0.20, *z*_r_ = −0.1, 0.45) snaps from the right buckling branch to the left during compression.

To demonstrate the design potential unlocked by using multiple slits, we experimentally realize two beams with many slits that exhibit targeted exotic behaviors. First, we realize a beam that is tristable at zero strain using six slits, and second, we realize a beam with a snapping transition from the right to the left buckling branch using three slits (Fig. 6).

To realize a slit-beam that is tristable at zero compressive strain, we combine the design ideas for realizing enhanced hysteresis (Fig. 5A) and for snapping in both branches (Fig. 5C). We first focus on the enhanced hysteresis and aim to create a beam that is bistable at zero strain, i.e., pushing ε_c_ to negative values (Fig. 6A and movies S8 and S9). To do so, we increase the slit depths to *s* = 0.8 and investigate a beam with three slits, at $z_{r}^{i}=\left(0.05\right)$ and $z_{l}^{i}=\left(-0.45, 0.4\right)$. Here, the snapping is initiated by the right slit, which then triggers the opening of both left slits simultaneously, leading to a postsnap configuration where the curvature is strongly concentrated near the slits so that the beam segments are nearly straight; this can be seen as a more extreme version of enlarged hysteresis range for the dual-slit beam (Fig. 5A). Here, this straightening of the beam segments lowers the collective closing of the three slits to negative strain values (ε_c_ < 0) and makes the right branch bistable at zero strain (Fig. 6A). To achieve tristability at zero strain, we induce three additional slits near $z_{l}^{i}=\left(-0.05\right)$ and $z_{r}^{i}=\left(0.45,-0.4\right)$, which satisfy the symmetry *z*_l_ → −*z*_r_ and *z*_r_ → −*z*_l_ as described above. Notice that this requires to slightly break the top-down symmetry of each set of three slits to avoid pairs of left and right slits at the same vertical position. While the slits in either of these two sets act cooperatively, the vertical alignment of the two sets induces strong anticooperative interactions between opposing pairs, and only one set is opened at each of the left and right snapped branches. Note that this approach also readily enables the creation of tristable beams with asymmetric branches by modifying slits from one of the sets, thereby allowing independent programming of each branch.

We lastly realize a beam with three slits that snaps from the right to the left buckling branch during compression. To program this response, we enhance the sequential snapping behavior observed in (iii) with an additional slit that allows the beam to overcome the energy barrier that separates the left and right branches (Fig. 6B and movie S10). The beam features three slits of depth *s* = 0.75, one cut from the left and two from the right at $z_{l}^{i}=\left(0.2\right)$ and $z_{r}^{i}=\left(0.45,-0.1\right)$. To systematically observe right buckling, we weakly break the left-right symmetry of the beam by introducing two small defects at the ends of the beams, which allows us to avoid the use of a static indenter. During compression, the beam first buckles right, after which the first slit-snapping instability that opens the bottom slit is triggered. The two other slits remain closed, and lowering the strain leads to a unsnapping transition analogous to a single slit beam (Fig. 6B).

However, further compression increases the curvature near the middle left slit, eventually producing a second slit-snap analogous to the phenomenon shown in (iii). This second instability changes the sign of the curvature near the top of the beam and triggers the opening of the top slit, which causes the beam to snap from the right to the left branch via an intermediate S-shaped configuration, reminiscent of the second buckling mode (*28*). The three slits are open in the final left branch configuration. When the strain is quasistatically removed, first, the bottom slit closes smoothly, resulting in a kink in the stability diagram, and then the two remaining slits collectively close at a lower strain, returning to the unbuckled configuration. From this state, the snapping sequence can be reinitialized by increasing the strain. These results highlight the broader potential of slit beams—with many slits—as a platform for encoding tailored multistability and directional instabilities.

## DISCUSSION

Endowing flexible structures with partial cuts that open and close as a result of global compressive stresses allow for a powerful collaboration between geometric and contact nonlinearities. For beams, we have uncovered the principle mechanisms that govern slit-enhanced snapping and demonstrated that it yields a combination of continuous buckling and discontinuous snapping under compression, large and tunable hysteresis cycles, multistability, and sequential behavior—all in a single slit beam. Interesting extensions include the use of slits for other geometries and the use of multiple interacting slit-snapping elements.

We close by briefly discussing three general areas of applications. First, snapping beams have been used to create materials that store and dissipate energy (*11*, *14*, *15*) and store information (*16*, *19*, *20*, *29*). As the number of stable states directly links with the energy and memory capacity, slits can be used to increase these capacities without changing the number of elements. Moreover, since energies and memories are often stored in buckled structures states (*15*, *19*, *25*, *29*, *30*), slit beams offer a simple way to tune or qualitatively modify their storage capacity (*31*, *32*).

Second, bistable elements have recently been used for information processing, with sequential driving encoding input and transitions between multistable states providing output—a concept that relies critically on hysteretic elements or hysterons (*19*, *20*, *29*, *31*–*39*). Mechanical implementations often rely on buckling under axial compression to store the information and lateral forcing to change it; however, slit beams allow to control buckling and snapping under the same type of forcing, opening up the possibility for simpler and more powerful driving protocols. Moreover, as slit-snapping concentrates deformations perpendicular to the driving, it facilitates the propagation of signals by interactions between neighboring elements (*19*, *30*). Last, as the interactions between multiple slits can both be cooperative and anticooperative, similar to the interactions needed to access complex sequences able to store memory and process information, slit-beams could form multi-bit building blocks for more advanced computing (*25*, *26*, *31*, *34*, *37*, *40*).

Third, physical intelligence in, e.g., soft robots or advanced metamaterials, often relies on instabilities and multistability, where switching between states can be triggered by interactions with the environment (*24*, *41*). We expect that our simple strategy with partial cuts will find applications in making soft robots more adaptive, faster, smarter, and easier to design.

## MATERIALS AND METHODS

### Manufacturing the beams

To manufacture slitted beams, we 3D print open-face molds to the beam’s desired dimensions, including a pin to create the hole. We then cast the molds using Smooth-On Mold Star 30 VPS rubber. A roughly incompressible rubber with a shore hardness of 30A, Young’s modulus *E* ≈ 738 kPa, and density ρ = 1120 kg/m^3^ (*42*). We allow the silicone to cure for at least 1 day before demolding. After demolding, we cut the slit using a custom device that features a scalpel attached to a linear slider rail. Last, we measure the thickness of the beam *W* with an accuracy of ±0.015 mm to correct for imperfections derived of the 3D printing of the mold.

### Uniaxial compression setup

We perform experiments in a custom build setup consisting of two parallel horizontal plates with controllable relative vertical distance in which we mount the beam and a horizontal indenter (fig. S1). We mount the beams using a pair of 3D printed, horizontally aligned clamps that tightly fit the support of the beams (fig. S1). We apply talc powder between the top and bottom parts of the slit of the beam, as well as between the indenter and the beam, to reduce the stickiness at these contact points.

The bottom plate position is fixed, while the top plate can be moved in the *y* direction with an accuracy of ±0.01 mm using an stepper motor connected to a computer. The plates are mounted on rails to ensure that the angle between plates is smaller than 0.06 mm/m. We calibrate the vertical distance between the plates using a copper beam of length 140 ± 0.03 mm; a dial indicator with sensitivity ±0.01 mm is used to check that the plates remain well calibrated during experiments.

### Clamping

We use clamped-clamped boundary conditions on the beams by casting them with two support blocks at each end that fit a pair of clamps attached to the plates. The clamps are aligned in the *x-z* plane by bringing them together before screwing them tightly to the plates. Calibration in *y* is achieved by pushing them toward the back of the clamps (fig. S1). Once a beam is clamped, it is firmly attached, and we do not see any drifting due to sliding after consecutive cycles (fig. S2). However, reclamping a sample can induce slight variations on the critical strains (fig. S3).

### Symmetry breaking

To control the initial buckling direction, we use a 3D printed indenter capped with a semicircular tip of radius 5 mm to set a finite midpoint deflection and explicitly break left-right symmetry. The indenter is attached to a manual linear stage that controls its *x* position with an accuracy of ±0.01 mm (fig. S1). The indenter pushes the beam laterally at height *L*/2, and we manually adjust its *x* position to set a minimum midpoint deflection *x*_in_. Experiments show that once the beam loses contact with the indenter, the effect of *x*_in_ on the beam configuration is minimal (fig. S2); in practice we use *x*_in_ of order 1 mm. When measures of both the right and left branches of a beam are performed, we first let the beam buckle spontaneously, and then we measure the other branch by using the indenter. This can result in small asymmetries near the buckling strain between the branches (Fig. 5).

### Data acquisition

A grayscale complementary metal-oxide semiconductor camera records the experiments at 3 Hz with a resolution of resolution of 3088 by 2064 pixels, yielding images with a pixel density of 5 px/mm. The beams feature tiny circular bumps that are painted white along their center line (fig. S1). We track the position of these dots along the beam with accuracy ±0.02 mm using a custom script based on the library opencv (*43*). We use the mean *x* distance between the two central dots and the neutral line to compute the midpoint lateral deflection *x*_m_.

### Numerical simulations

We complement the experiments with FEM simulations to tackle two limitations of the experiments; First, numerical simulation allow us to avoid experimental issues due to reclamping. Second, the idealized material used in the simulation eliminates the material-induced hysteresis steaming from material creep (Fig. 2A) and proves that slit-snapping arises from geometry of the beam rather than material properties (fig. S5). We perform simulations in the software package Abaqus, using the Dynamic/Explicit solver, 2D geometry with plain stress, a Neo-Hookean material with a Poisson ratio ν = 0.49, Young’s modulus *E* = 0.78 MPa, and sufficient damping to avoid oscillations.
