# Supplementary material for: Hysteretic slit-snapping and multistability in buckled beams with partial cuts
Source: Sci Adv. 2026 Feb 13;12(7):eaeb9750. doi: 10.1126/sciadv.aeb9750 (PMC12904158; doi:10.1126/sciadv.aeb9750)
Supplement: Supplementary file 1 — Supplementary Text Figs. S1 to S8 Legends for movies S1 to S10 [file sciadv.aeb9750_sm.pdf]

Science Advances
AAAS

Supplementary Materials for

Hysteretic slit-snapping and multistability in buckled beams with partial cuts

Bernat Durà Faulí *et al.*

Corresponding author: Martin van Hecke, mvhecke@gmail.com

Sci. Adv. **12**, eaeb9750 (2026)
DOI: 10.1126/sciadv.aeb9750

The PDF file includes:

Supplementary Text
Figs. S1 to S8
Legends for movies S1 to S10

Other Supplementary Material for this manuscript includes the following:

Movies S1 to S10

Single offset slit

Since multiple slits require offsetting of at least one from the beam's vertical midpoint, we examine the effect of the vertical position $z_r := Z_r/L$ of a single right slit (Fig. 4 and Fig. 5). We find that while the buckling threshold is insensitive to slit position, the opening and closing strains increase monotonically with z_r; off-center slits require larger strains to open (Fig. S4). In addition, the hysteresis loop gradually shrinks and appears to close as $|z_r|$ approaches 0.19; for $|z_r| > 0.19$, snapping is no longer observed (Fig. S6B). These trends stem from the variation of the beam curvature with z_r. In good approximation, the shape of a right-buckled beam follows $x(z) \propto sin(2\pi z/L)$, and features inflection points at $z = \pm 0.25$, so that the curvature is maximal for $z_r = 0$ and becomes negative for $|z_r| > 0.25$. Hence, stresses across a right slit in a right buckled beam can only become tensional in the center segment of the beam, and for our particular beam geometry, a right slit only leads to right snapping when $|z_r| \lesssim 0.19$ (Fig. S4).

Truss model

We numerically find solutions to the spring model by looking at it's potential energy, which can be written as the sum of the potential energy of the springs:

$$E = k_0 u_l^2 + \kappa_\alpha \alpha^2 + \frac{1}{2}\kappa_\theta \theta^2 \tag{1}$$

where

$$u_l = l_0 - \sqrt{x^2 + y^2} \tag{2}$$

$$y = l_0(1 - \varepsilon) - l_H \sin\theta \tag{3}$$

$$\alpha' = \alpha - \pi/2 = \theta + \text{arctan2}(y, x) - \pi/2 \tag{4}$$

Without loss of generality we take $l_s = 1$ and $k_s = 1$ and write the dimensionless energy as

$$\hat{E} = \hat{u_l}^2 + \hat{\kappa}_\alpha \alpha'^2 + \frac{1}{2}\hat{\kappa}_\theta \theta^2 \tag{5}$$

where $\hat{u_l} = 1 - \sqrt{x^2 + y^2}$ is the strain of l_s and $y = (1 - \varepsilon) - \lambda \sin\theta$, where $\lambda = l_H/l_s$.

We are left with three parameters to be determined, l_H, κ_α and κ_θ, the same number of critical values in slit snapping, ε_b^{model}, ε_o^{model}, ε_c^{model}. By crossing them in the right order, is possible to control each of the critical strains with a single model parameter, providing the model parameters of a physical interpretation. The buckling threshold in S_c is determined only by κ_α, as the contributions to the potential energy from both l_H and κ_θ vanish for $\theta = 0$. Therefore, we choose a value for κ_α that accurately reproduces ε_b in physical beams. Once κ_α is fixed, the structure snapping strain is determined by l_H. Remarkably, choosing $l_H/l_s = \lambda$, yields a good accurate prediction of ε_o, underscoring the geometric origin of slit-snapping (Main Fig. 3). Finally, with κ_α and l_H fixed, the parameter κ_θ remains as a free variable used to match the experimentally observed critical snapping strain ε_c. Physically, κ_θ encodes the depth of the slit: in the limit $\kappa_\theta \to \infty$, the hinge remains rigid, emulating an unslit beam. Decreasing κ_θ mimics the effect of increasing the slit depth s, thereby reducing ε_c, in agreement with experimental observations.

The pathway of this system in response to driving can be found by tracing the local extrema of the potential as a function of ε (Fig. S8). This requires taking into account the unilateral constraint imposed by the slit: $\theta \geq 0$. We solve the stability of the complete system by considering two related systems: the unconstrained system S_f, in which θ is free and thus the slit can open and even self-intersect; and the system S_c, where $\theta = 0$ remains fixed on the boundary, and the slit remains closed. To solve the stable and unstable states of S_f, we calculate the potential energy E on a sufficiently dense grid in x and θ, and find the local minima and saddle points by intersecting the numerical contour curves of $\partial E/\partial x$ and $\partial E/\partial \theta$ at each ε. For system S_c, we find the roots of the numerically determined $\partial E/\partial x$ at each ε.

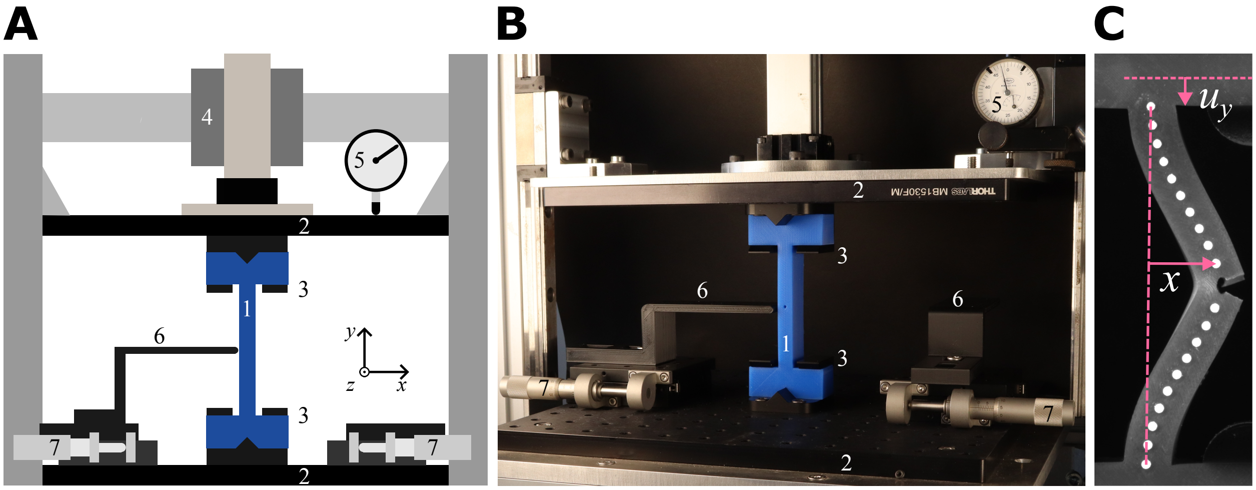

Figure S1: **Experimental set-up**. **(A-B)** beam (1) is clamped between two parallel plates (2) using custom steel clamps (3). A triangular dent on the clamps fit tightly the beams support block to align both beam ends in the x-direction. Alignment in the z direction is achieved by pushing the beam's supports towards the back end of the clamps. A stepper motor (not featured) drives the position of the central bearing block (4), which controls the vertical position of the top plate. A height gauge dial (5) allows to keep track of the neutral position during and in between experiments. A 3D printed indenter (6) is mounted on a micrometer controlled horizontal linear stage (7) that sets its x-position. **(C)** Computer image used for tracking. The mid beam deflection x is computed tracking the white painted dots along the beam's profile.

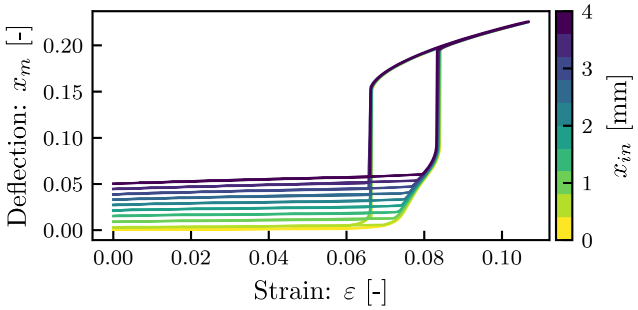

Figure S2: **Effect of the indenter.** Experimental response of a slit beam with $\lambda = 0.15$, $s = 0.8$ and $h = 1$ to consecutive driving cycles where the indenter position x_{in} is increased by 0.5 mm after each cycle. The effect of the indenter on the relevant parts of the curves is negligible.

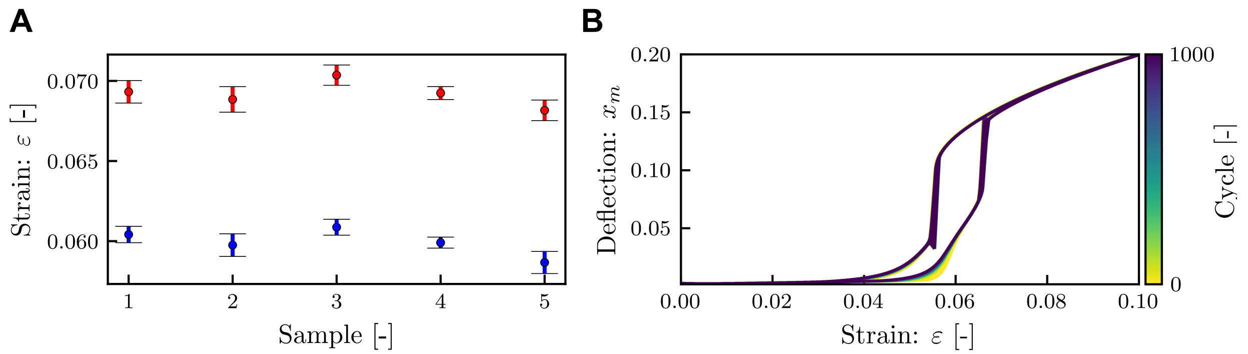

Figure S3: **Slit-snapping reproducibility. (A)** Critical strains ε_o and ε_c for five different samples with $\lambda = 0.133$, $s = 0.6$, $H = 2mm$. Error-bars show the standard deviation of the mean for five independent measurements, where the beam is re-clamped. **(B)** Slit snapping is robust and consistent during 1000 driving cycles. Results for sample 5 in panel A.

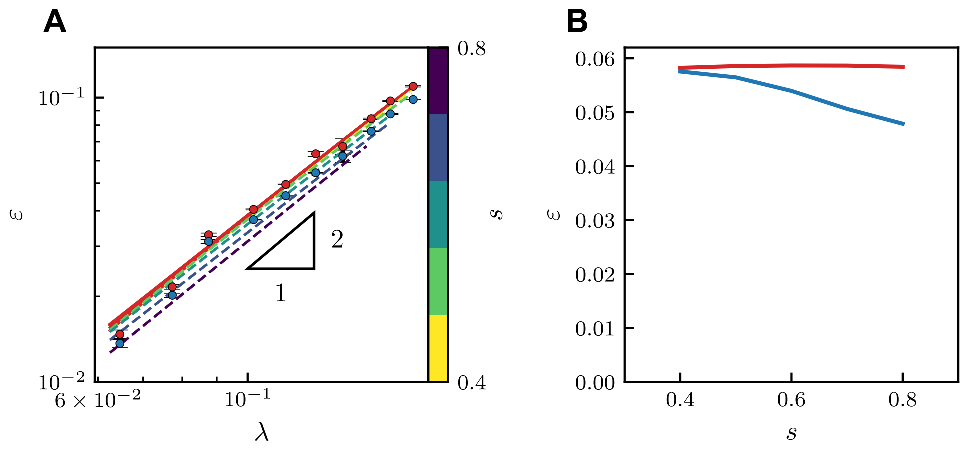

Figure S4: $\boldsymbol{\varepsilon_o}$ **and** $\boldsymbol{\varepsilon_c}$ **dependence on** λ **and s. (A)** Numerical (lines) and experimental data (dots) of the evolution of ε_o (red) and ε_c (colorbar) for λ values in the range $\lambda = (0.0625, 0.175)$ and $s = (0.4, 0.8)$ for numerics and s=(0.6) for experiments. Error bars show the standard deviation of the mean of five measurements where the beam is re-clamped between them. **(B)** Numerical results of the evolution of ε_o (red) and ε_c (blue) with the slit depth s for a beam with fixed $\lambda = 0.125$.

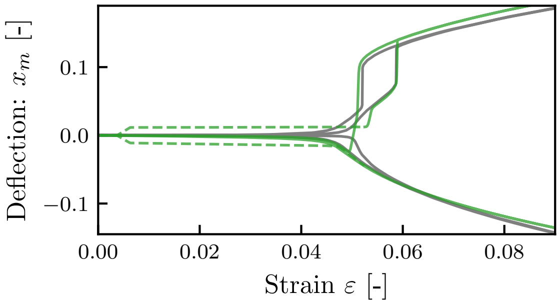

Figure S5: **Numerical bifurcation diagram (A)** Numerically obtained bifurcation diagram (green) for a beam with the same dimensions as the sample in (Fig. 2A) in the main text; λ = 0.125, s=0.6, H=2mm. Dashed lines correspond to beams in contact with an indenter, that pushes the beam left or rightwards. In gray, the experimentally obtained bifurcation diagram also shown in (Fig. 2A) in the main text

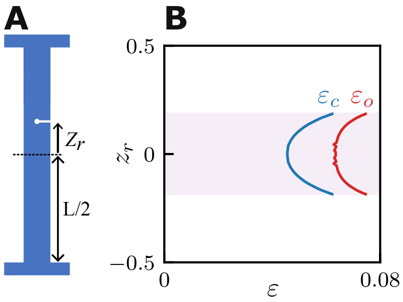

Figure S6: **Slit-snapping of off-center slits** (**A-B**) For a beam with a single-slit, its vertical position $z_r = Z_r/L$ modulates the opening and closing strains (ε_o (red), ε_c (blue)), and for $|Z_r| > 0.19$, no snapping is observed.

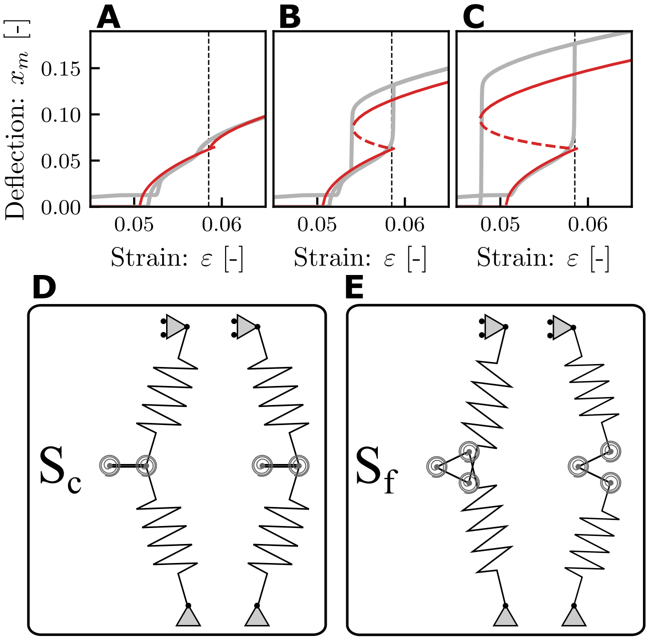

Figure S7: **Spring model accurately reproduces the regimes of small and big slits and buckled configurations of the spring system in the closed and free cases.** Spring model bifurcation diagrams (red) over the Abaqus numerically obtained bifurcation diagrams with Abaqus (gray) of the main text. Constant $l_H/l_0 = \lambda = 0.125$ and $\kappa_\alpha = 0.048$. (**A**) $\kappa_\theta = 10\kappa_\alpha$, (**B**) $\kappa_\theta = 3\kappa_\alpha$, (**C**) $\kappa_\theta = 1.8\kappa_\alpha$ (**D-E**) Sketch of the buckled spring configuration in the constrained case, $\theta = 0$. **(D)** Sketch of the buckled spring configuration in the constrained case S_c with $\theta = 0$. **(E)** Sketch of the buckled spring configuration in the free case S_f. The rigid bars of the hinge self intersect when the system is in the left branch.

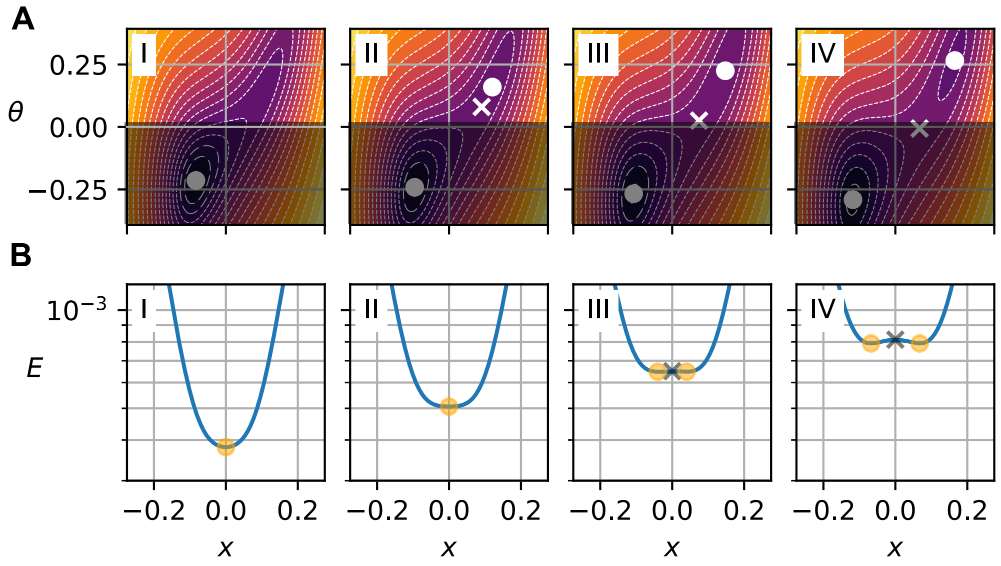

Figure S8: **Truss model energy landscape** (A) The potential energy of the truss spring-system as a function of θ and x ($\hat{\kappa}_\alpha = 0.0456$, $\hat{\kappa}_\theta = 0.113$ and $l_H = 0.126$) for four values of ε= [0.039,0.045,0.051,0.057]. The white dots represent local minima, and the crosses represent saddle points. The lower half $\theta < 0$ is shaded darker to represent non-admissible regions where there is self-intersection in C_f. The white contour lines correspond to lines of equipotential. (B) The potential energy of the system as a function of x at constant $\theta = 0$, captures the buckling bifurcation for in the constrained configuration C_c. The orange dots represent local minima and the crosses represent maxima.

Movie legends

Movie S1: Right buckling of a beam with a single slit. Beam geometry: $\lambda = 0.125$, $z_r = 0$, s=0.6, H=2 mm. Shown at 100x speed. Video corresponding to figure 2A in the main text.

Movie S2: Left buckling of a beam with a single slit. Beam geometry: is $\lambda = 0.125$, $z_r = 0$, $s = 0.6$, $H = 2mm$. Shown at 100x speed. Video corresponding to figure 2A in the main text.

Movie S3: Large hysteresis in a dual slit beam. Beam geometry: $\lambda = 0.124$, $z_l = 0.45$, $z_r = 0$, $s = 0.75$, $H = 1mm$. Shown at 20x speed. Video corresponding to figure 5A in the main text.

Movie S4: Snapping in a dual left-slit beam. Beam geometry: $\lambda = 0.124$, $z_l = 0.475$, $z_l = 0.-475$, $s = 0.75$, $H = 1mm$. Shown at 20x speed. Video corresponding to figure 5B in the main text.

Movie S5: Right snapping of a dual slit beam. Beam geometry: $\lambda = 0.124$, $z_l = -0.05$, $z_r = 0.05$, $s = 0.75$, $H = 1mm$. Shown at 20x speed. Video corresponding to figure 5C in the main text.

Movie S6: Left snapping of a dual slit beam. Beam geometry: $\lambda = 0.124$, $z_l = -0.05$, $z_r = 0.05$, $s = 0.75$, $H = 1mm$. Shown at 20x speed. Video corresponding to figure 5C in the main text.

Movie S7: Sequential snapping in a dual slit beam. Beam geometry: $\lambda = 0.125$, $z_l = 0.2$, $z_r = -0.1$, $s = 0.75$, $H = 1mm$. Shown at 20x speed. Video corresponding to figure 5D in the main text.

Movie S8: Right snapping of a tristable beam at zero strain. Beam geometry: $\lambda = 0.1365, s = 0.8, z_l = -0.45, -0.05, 0.4, z_r = -0.4, 0.05, 0.45$. Shown at 20$x$ speed. Video corresponding to figure 6A in the main text.

Movie S9: Left snapping of a tristable beam at zero strain. Beam geometry: $\lambda = 0.1365, s = 0.8, z_l = -0.45, -0.05, 0.4, z_r = -0.4, 0.05, 0.45$. Shown at 20$x$ speed. Video corresponding to figure 6A in the main text.

Movie S10: Right-left snapping of a multi slit beam. Beam geometry: $\lambda = 0.126, s = 0.75, z_l = 0.20, z_r = -0.1, 0.45$. Shown at 20$x$ speed. Video corresponding to figure 6B in the main text.
